# Morphogenetic (Mucin Expression) as Well as Potential Anti-Corona Viral Activity of the Marine Secondary Metabolite Polyphosphate on A549 Cells

**DOI:** 10.3390/md18120639

**Published:** 2020-12-14

**Authors:** Werner E. G. Müller, Meik Neufurth, Shunfeng Wang, Rongwei Tan, Heinz C. Schröder, Xiaohong Wang

**Affiliations:** 1 ERC Advanced Investigator Grant Research Group at the Institute for Physiological Chemistry, University Medical Center of the Johannes Gutenberg University, 55128 Mainz, Germany; mneufurt@uni-mainz.de (M.N.); shunwang@uni-mainz.de (S.W.); hschroed@uni-mainz.de (H.C.S.); 2 Shenzhen Lando Biomaterials Co., Ltd., Building B3, Unit 2B-C, China Merchants Guangming Science Park, Guangming District, Shenzhen 518107, China; tanrw@landobiom.com

**Keywords:** mucin, polyphosphate, hydrogel, ATP, innate immunity, SARS-CoV-2 spike protein, human alveolar cells

## Abstract

The mucus layer of the nasopharynx and bronchial epithelium has a barrier function against inhaled pathogens such as the coronavirus SARS-CoV-2. We recently found that inorganic polyphosphate (polyP), a physiological, metabolic energy (ATP)-providing polymer released from blood platelets, blocks the binding of the receptor binding domain (RBD) to the cellular ACE2 receptor in vitro. PolyP is a marine natural product and is abundantly present in marine bacteria. Now, we have approached the in vivo situation by studying the effect of polyP on the human alveolar basal epithelial A549 cells in a mucus-like mucin environment. These cells express mucins as well as the ectoenzymes alkaline phosphatase (ALP) and adenylate kinase (ADK), which are involved in the extracellular production of ATP from polyP. Mucin, integrated into a collagen-based hydrogel, stimulated cell growth and attachment. The addition of polyP to the hydrogel significantly increased cell attachment and also the expression of the membrane-tethered mucin *MUC1* and the secreted mucin *MUC5AC*. The increased synthesis of MUC1 was also confirmed by immunostaining. This morphogenetic effect of polyP was associated with a rise in extracellular ATP level. We conclude that the nontoxic and non-immunogenic polymer polyP could possibly also exert a protective effect against SARS-CoV-2-cell attachment; first, by stimulating the innate antiviral response by strengthening the mucin barrier with its antimicrobial proteins, and second, by inhibiting virus attachment to the cells, as deduced from the reduction in the strength of binding between the viral RBD and the cellular ACE2 receptor.

## 1. Introduction

Human viruses are obligate cell parasites that infect their target cells/tissues via specific portals of entry. They predominantly interact with the host epithelium, which lines both the outer surface and the inner cavities of the body. Among them is the mucosal epithelium, which is covered by a protective layer, the mucus. This epithelium spans the respiratory tract, the gastrointestinal tract, and the genital tract. This cell barrier can be bypassed if the viruses can directly access the internal sites within the tissue, e.g., after biting of an insect or an animal, or through transplantation of a virally infected organ. Cell damage or cell death after a virus infection results in a shutdown of cellular macromolecular synthesis as a consequence of the competition of viral promoters and transcriptional factors with their cellular counterparts, which leads to an abolition of the interferon defense mechanisms and also to a diversion, a rewiring of energy metabolism. Since the cellular energy channeling is essential for both cell survival and viral propagation, the direction of energy dissipation and distribution is decisive for the stability of the host and the infectious agent [1]. A disruption of cellular metabolic energy supply can lead to apoptosis of the host cells [2] or autophagy [3].

The epithelium of the nasopharyngeal region and the lungs represents the largest non-keratinized moisty surface of the human body that is exposed to the environment and is challenged by huge amounts of bacteria and viruses present in the inhaled air and aerosols. These include RNA viruses such as coronaviruses, rhinoviruses, respiratory syncytial virus, and influenza virus that invade the lungs. In turn, these tissue cell layers are provided with efficient innate immune defense systems that recognize these viruses and respond to them with downstream signaling cascades (reviewed in: [4]).

Two arms of host cells/organisms fight against viral propagation, first the adaptive immune response and second, the highly conserved immediate/early innate immune response [5,6]. While the lymphocytes of the adaptive immune system provide versatile and specific means of defense and protect against re-infection with the same pathogen, the neutrophils and thrombocytes (platelets) of the innate immune system form the first line of defense against many microorganisms and viruses [7]. The innate antiviral immune response includes the expression of interferons, cytokines, chemokines, and the activation of cell death pathways and might be beneficial for the host following virus infection; these reactions are energy-intensive [8,9]. In the epithelium of the nasopharyngeal region and of the lungs, the first barrier against invading pathogens is the mucus layer [10,11]. Additionally, the novel coronavirus (2019-nCoV; severe acute respiratory syndrome coronavirus 2 (SARS-CoV-2)) enters the host via the nasal and bronchial epithelium [12]. There, SARS-CoV-2 enters the cells by binding of its spike (S) protein to the specific cellular receptor, the angiotensin-converting enzyme 2 (ACE2) [13]. The viral entry depends on the processing of the S-protein by the proteases TMPRSS2 and cathepsin B/L. These components are present together with the ACE2 in both the nasal and bronchial epithelium [14]. The mucus with its mucin component(s) can efficiently trap SARS-CoV-2 with its size of ~100 nm [15]. This “mucoadhesion” is based on the binding interactions of mucin with the diffusing particles [16]. The major gel-forming components of the mucus are the mucins with their large glycoproteins with sizes of >100 kDa, which carry carbohydrate groups such as *N*-acetylgalactosamine, fucose, galactose, and sialic acid. It has not yet been clarified whether the antiviral effect of mucin is restricted to the mechanical barrier forming properties of mucin alone or whether it also involves a biochemical intracellular defense signaling pathway [15]. Given the mucoadhesive properties of mucin, it can be expected that the mucus may act as a basket for non-covalent association of cell signaling molecules such as interleukins [17] or other small molecules [18]. Needless to mention is that the mucins contain antimicrobial proteins, such as lysozyme and lactoferrin [19], as well as the gp340, a salivary agglutinin [20], which displays antiviral activity. Interestingly enough is that the mucins are compartmented and allow a targeted attack of the microbes by, e.g., the skin-antimicrobial peptide, β-defensin 2 [21]. Furthermore, it can be assumed that the genuine mucins, especially the cell-associated mucin species, like other glycoconjugates, also elicit bioactive signals via their cytoplasmic regions [22,23]. The outer shell of the COVID-19 viruses is resistant and hard [24,25]. A solution to break it is not yet available [26]. Perhaps the cationic antimicrobial fragment of MUC7, RKSYKCLHKRCR, which possesses distinct antimicrobial activity [27], is an adequate approach.

It has not yet been studied whether the composition and organization of the airway surface liquid layer on the epithelium of the respiratory tract is altered during the corona virus infection. Under physiological conditions this zone comprises two layers; the mucus layer, contacting the air, which lies above the periciliary layer in which the surface cilia and the corresponding cells are lined up. The periciliary layer is less viscous than the surrounding gel layer above it. The gel layer is composed of the secreted mucins, MUC5AC and MUC5B, while the periciliary layer is formed from the membrane-tethered mucins MUC1, MUC4, and MUC16, which are present on the surfaces and microvilli of the ciliated and the secretory cells (reviewed in: [28]).

In the approach presented here, the expression of mucins in the well-characterized human lung carcinoma/alveolar cell line A549, used as model cell system, which is representative for alveolar type 2 cells [29], has been studied. This cell line inducibly expresses the different *MUC* genes [30,31] and comprises the most important secretagogue of the surface epithelium, the machinery for the formation and release of ATP as well as its effect on the membrane-associated P2Y_2_ receptors also acting on the apical membrane of A549 cells [32]. In addition, these cells comprise on their surfaces the two enzymes involved in the extracellular ATP generation cycle, alkaline phosphatase (ALP) and adenylate kinase (ADK) [33,34,35]. The exocytosis of ATP is dependent not only on hypotonic swelling of the alveolar A549 cells but also on a synergistic autocrine/paracrine effect of co-released uridine and adenosine nucleotides [36]. In turn, the buffering of the ATP level in and around of the alveolar type 2 cells, like during hypoxia, is maintained by the exocytosis pathway, which is controlled by the extracellular ATP generation cycle [37]. The basic ATP levels around A549/alveolar type 2 cells in vitro measure <50 pmol/10^6^ cells [38].

Evidence suggests that SARS-CoV-2 is spread primarily through saliva droplets or discharge from the nose [39] the primary entry portal of the virus. Furthermore, it has been proposed that the virus can enter lungs and oral tissue directly via the cellular ACE2 receptor since patients are complaining about symptoms such as dry mouth and hypogeusia. Consequently, saliva the common and transient medium for virus transmission is centrally important for SARS-CoV-2 infection. Until now, it has not been confirmed that dry cough is a further sign of corona virus infection [40], and it also remains to be studied if the level of mucin in the saliva is correlated with the severity of the infection, as suggested for the human immunodeficiency virus [41].

It has been outlined that the level of extracellular ATP is adjusted by a physiological polymer, by polyphosphate (polyP) (reviewed in: [33]), which is abundantly present in any type of cells, especially in the blood platelets [42]. PolyP is synthesized intracellularly in close association with mitochondria and then exported into the extracellular space by exocytosis following platelet activation [43,44]. The polyP content in the saliva has not been determined. However, both bacteria —a likewise rich reservoir for polyP [45]—and inflammatory cells, including the platelets, can also colonize under the healthy watery saliva and thick mucus [11]. The surfaces of the human airway epithelia expose the nonspecific ALP [46], which hydrolyzes polyP and releases metabolic energy that contributes to the phosphorylation of AMP to ADP [44]. The subsequent phosphorylation of ADP to ATP is catalyzed by the ADK, likewise an ecto-enzyme present on the human airway epithelial cells [47]. Low platelet counts, correlated with thrombocytopenia, are signs of SARS-CoV-2 infection [48] and certainly will result in a reduced polyP supply.

Recently, we described that polyP blocks the binding of the receptor binding domain (RBD) of the SARS-CoV-2 S-protein to the cellular ACE2 receptor in vitro [49,50]. Surprisingly, the inhibition is measured already at a low concentration of 0.1 µg/mL [50], which is lower than that in the circulating blood with 1 to 3 μg/mL [42]. In order to elucidate the proliferation und functional activity of A549 cells under close-to-normal conditions, a hydrogel was fabricated from submaxillary gland mucin and collagen and used as a matrix for the in vitro studies.

PolyP is abundantly present in any cells and occurs in large amounts in marine organisms, such as in the marine cyanobacterium *Synechococcus* sp. as 30–70 nm nanoparticles [51], or in the sulfur bacteria *Beggiatoa* or *Thiomargarita* [52], and also in sponges in particles ranging from 0.5 to 3 μm [53].

A549 cells were incubated on collagen-based hydrogels and found to readily attach to the surfaces and cause an increased expression of high levels of the mucin genes, *MUC1* and *MUC5AC*. In previous studies, it has been established that mucin gene expression can be induced by cytokine and progesterone and in epithelial cells by inflammatory cytokines [54].

Since the previously observed morphogenetic activities of polyP [33] can be correlated with the intra-and/or extracellular ATP level, the effects of polyP on A549 cells in the hydrogel environment were also determined in the present study. Again, an increase in the release of ATP after exposure to polyP was found. This effect is inhibited by both the ADK inhibitor Ap5A and the ALP inhibitor levamisole [55,56], we conclude that cells grown on the mucin hydrogel process the integrated polyP and convert the energy released to ATP. At first, ALP hydrolyzes the polymer under release of phosphate (P_i_) [57] and metabolically usable Gibbs free energy (ΔG) that are used for converting AMP to ADP and finally via the ADK to ATP. After the addition of polyP to mucin this glycoprotein, acting mechanically as a first barrier in the saliva against invading bacteria and viruses, gains morphogenetic potential and induces *mucin* gene expression via ATP generation. In turn, mucin in combination with polyP acts—like the interferons [58]—as a signaling molecule by stimulating the innate antiviral responses. This effect is amplified by the direct antiviral effect of polyP on the level of binding of SARS-CoV-2 to the host cell. Therefore, polyP holds a dual antiviral innate defense action potential; first, it blocks viral attachment and second, it boosts the antiviral, innate immune function of the airway mucosal barrier.

Finally, data are presented showing that polyP sensitively inhibits the interaction of the RBD with the cellular ACE2 receptor, and this effect is also observed in the presence of mucin.

## 2. Results

### 2.1. Collagen-Based Mucin–PolyP Hydrogel

**Figure 1:**
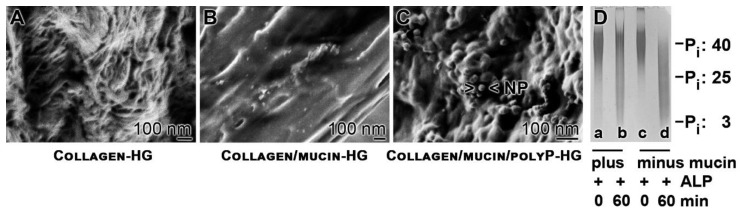
The polymer polyP. (**A**–**C**) Hydrogel formation; Scanning electron microscopy (SEM). (**A**) Collagen-HG, (**B**) Collagen/mucin-HG, and (**C**) Collagen/mucin/polyP -HG. In (**C**), nanoparticles (NP) are seen that are formed with polyP in the presence of Mg^2+^. (**D**) Size separation of Na-polyP by 7 M urea/25% polyacrylamide gel electrophoresis. As markers to the Na-polyP sample used in the study (~40 P_i_ units), Graham’s salt (25 P_i_) and trimeric Na-polyP_3_ (3 P_i_ units) were used. Na-polyP was incubated in the presence (plus; lanes a and b) or absence (minus; c and d) of mucin and in the presence of alkaline phosphatase (ALP) for 0 min (a and c) or 60 min (b and d).

The hydrogel-forming potential of collagen was exploited to provide a suitable scaffold for the A549 cells to grow. At pH 3.6, the physically crosslinked collagen shows still individual fibrils of a diameter of ~50 nm ***(***Figure 1A). After adjusting the material to pH 7.4 the collagen hydrogel (Collagen-HG) is formed, which presents an almost smooth surface. MgCl_2_ was added to the sample in order to provide also a comparable matrix for the cells if mucin and polyP are added. The addition of mucin (final concentration of 100 µg/mL) together with 10 mM MgCl_2_ to the collagen sample at pH 3.6, followed by an increase to pH 7.4, did not change the texture—smooth surface—of the collagen/mucin-hydrogel (Collagen/mucin-HG) matrix (Figure 1B).

The polymer polyP was added to the Collagen/mucin-HG at concentrations between 0 and 100 µg/mL under formation of a collagen/mucin/polyP-hydrogel (Collagen/mucin/polyP-HG). In order to compensate the negative charges of the polyP, divalent cations (MgCl_2_) were added. The Na^+^-salt of polyP (added to the sample) is soluble. After addition of divalent cations, polyP forms more insoluble salts, especially at more alkaline conditions (>pH 9.5). Under the conditions used here and in the presence of mucin, polyP forms after exposure to Mg^2+^ nanoparticles of a size between 50 and 100 nm (Figure 1C). By thermogravimetric analysis, about 80 to 90% of the polyP added to the hydrogel remained in the matrix.

### 2.2. Increased Resistance of PolyP Against Alkaline Phosphatase in the Presence of Mucin

The polymer was separated by polyacrylamide gel electrophoresis (PAGE). Using 15% (or–where mentioned–25%) polyacrylamide/7 M urea gels the Na-polyP sample, studied here, run as a fairly sharp band at ~40 P_i_ units with a smear within the range of 20 to 60 P_i_ units (Figure 1D). The standard sample (100 µg/mL) with a chain length of 40 P_i_ was incubated in the absence or presence of mucin (100 µg/mL) with ALP (5 µg/mL) for 0 min or 60 min. Then, aliquots were size separated. The samples at the beginning of the incubation (time 0 min) run at the same front, irrespective if mucin was present or not (Figure 1D; lanes a and c). However, the sample supplemented with mucin and incubated for 60 min showed a much lower degradation, compared to the one incubated in the absence of mucin. In the presence of mucin, still higher molecular-weight polyP (around 40 P_i_ units) can be resolved (Figure 1D; lane b), while the sample in the absence of mucin was degraded down to the trimer (Figure 1D; lane d).

### 2.3. Fourier-Transform Infrared Spectroscopy

**Figure 2:**
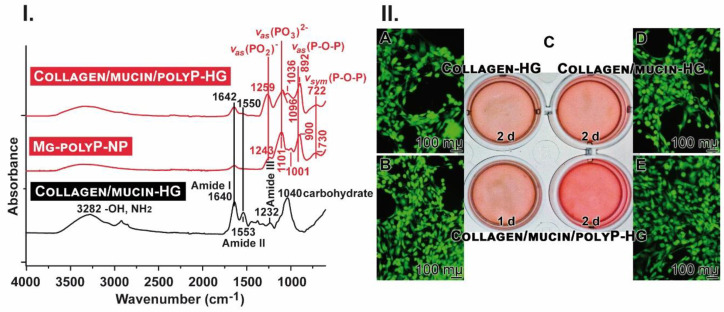
The hydrogels and cells growing on them. (**I**) The FTIR spectral characteristics for the two hydrogels Collagen/mucin-HG and Collagen/mucin/polyP-HG in comparison to Mg-polyP-NP. In the mucin gel, the characteristic polyP signals (PO_2_, PO_3_ and P–O–P) are missing. (**II**) Growing of A549 cells onto the gels in 24-well plates (**C**) and staining of the cells with calcein; (**A**) Collagen-HG (incubation for 2 days), (**B**,**E**) Collagen/mucin/polyP-HG (1 day or 2 days), or (**D**) Collagen/mucin-HG (2 days).

The Fourier-transform infrared spectroscopy (FTIR) spectra of Collagen/mucin-HG, Collagen/mucin/polyP-HG and, as reference for polyP, of Mg-polyP-NP were recorded (Figure 2I). The Collagen/mucin-HG spectrum shows three main characteristic absorption bands within the glycosylated protein structure [59]. The first region is the broad band centered at 3282 cm^−1^ that refers to the stretching vibration of the amino groups (–NH_2_) and the hydroxyl group (–OH). The second one at 1640 cm^−1^ is attributed to C=O stretching vibration of amide I band, while the amide II band representing N–H bending vibration and C–N stretching vibration is observed at 1553 cm^−1^. The third region at 1232 cm^−1^ is assigned to the stretching vibrations due to the C–O bonds in the carboxyl group and the strong band centered at 1040 cm^−1^ is due to mucin carbohydrate sidechains.

The spectrum of Mg-polyP-NP shows the characteristic phosphate bands at 1243 cm^−1^ and 1101 cm^–1^, which are assigned to the asymmetric stretching vibrations in the PO_2_ and the PO_3_ groups, respectively. The symmetric stretching vibration of the PO_3_ group is observed around 1001 cm^−1^, while the asymmetric and symmetric stretching vibrations of the P–O–P bridge are seen at 900 cm^−1^ and 730 cm^−1^, respectively. In addition, the broad bands at 3330 cm^−1^ and 1638 cm^–1^ are attributed to the stretching vibration and bending of the –OH groups.

The spectrum of Collagen/mucin/polyP-HG sample shows the characteristic bands of both Mg-polyP and mucin, in which the C=O stretching vibration of amide I band (1640 cm^−1^) is pronounced at 1642 cm^−1^. In addition, the amide II band (1553  cm^−1^) is observed as small shoulder at 1550 cm^−1^. The characteristic polyP bands (Mg-polyP-NP) are observed at 1259 cm^−1^, 1096 cm^−1^, 1036 cm^−1^, 892 cm^−1^, and 722 cm^−1^. The changes within amide I and II as well as some polyP bands are attributed to the interactions between both mucin and polyP through the Mg^2+^ ions.

### 2.4. Attachment of Cells to the Hydrogels

Human lung cells, A549, were seeded onto either Collagen hydrogel, Collagen/mucin-HG, or onto Collagen/mucin/polyP-HG. The gels formed a tight layer also with the edge of the wells (Figure 2(IIC)). Already, after an incubation for 2 days, it becomes apparent by eye-inspection that the density of the cells growing onto Collagen/mucin/polyP-HG is higher, compared to Collagen/mucin-HG or to Collagen-HG. Even after a 1 day culturing onto Collagen/mucin/polyP-HG, the density of the polyP-containing hydrogel is as high as the ones onto the polyP-free matrices. The cells were growing readily onto the three hydrogels, as judged by calcein staining. However, the cell density after 2 days is higher onto Collagen/mucin/polyP-HG, compared to the ones onto Collagen-HG or onto Collagen/mucin-HG (Figure 2(IIE), compared to A and D). Even after 1 day, the cell density onto Collagen/mucin/polyP-HG is high (Figure 2(IIB)).

### 2.5. Stimulation of Cell Growth on Collagen–Mucin Hydrogel

**Figure 3:**
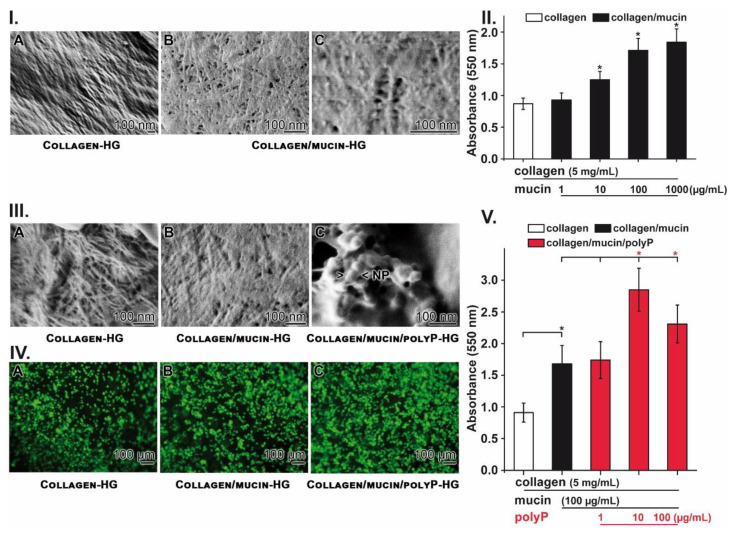
Viability and growth of A549 cells on the hydrogels. (**I**) Surface of (**A**) Collagen-HG and (**B**,**C**) Collagen/mucin-HG; SEM. (**II**) Growth of A549 cells during a 48-h incubation period. The viability was determined by the MTT assay system. (**III**) Comparative photo image of the surface of (**A**) Collagen-HG, (**B**) Collagen/mucin-HG, and (**C**) Collagen/mucin/polyP-HG; SEM. In the polyP-containing matrix, nanoparticles of sizes between 50 and 100 nm have been formed from polyP and MgCl_2_. (**IV***)* Already after microscopical inspection of the cells, after staining with calcein, a strong difference in the density of the cells between cultures onto Collagen-HG (**A**) and Collagen/mucin-HG (**B**) in comparison to Collagen/mucin/polyP-HG (**C**) is apparent. (**V**) Growth of A549 cells on the three different matrices during a 48-h incubation period. The cells were seeded onto Collagen-HG (collagen: 5 mg/mL) alone or supplemented with mucin (100 µg/mL) or additional also with polyP (1 to 100 µg/mL). After an incubation period of 48 h, the viability was assessed using the MTT assay. The values are expressed as absorbance values at 550 nm. The number of parallel experiments was 10. Data are means ± SD (* *p* < 0.05).

Growth/viability of A549 cells was studied first in the absence of polyP on both Collagen-HG (Figure 3(IA)) and Collagen/mucin-HG (Figure 3(IB,C)). Supplementation of collagen with mucin results in a coating of the collagen fibrils (Figure 1 and Figure 3(IA)) with mucin glycoproteins. By this, the collagen fibrils glue together (Figure 1B or Figure 3(IB,C)).

The growth of the cells was quantitated with the MTT (3-(4,5-methylthiazol-2-yl)-2,5-diphenyl-tetrazolium bromide) assay system on Collagen-HG, containing 5 mg/mL of collagen. After an incubation period of 48 h the cell density increased from 1 × 10^4^ cells/mL to 4.29 × 10^4^ cells/mL. The addition of mucin (Collagen/mucin-HG) with a final concentration of 10 µg/mL caused a significant increase in growth by 60.8% (to 6.9 × 10^4^ cells/mL). The addition of 100 µg/mL of mucin even stimulated the growth by 100.1% (to 8.58 × 10^4^ cells/mL); Figure 3II.

In the following series of experiments, the growth of A549 cells was studied, again on both Collagen-HG (Figure 3(IIIA)) and Collagen/mucin-HG (Figure 3(IIIB)) and finally also on Collagen/mucin/polyP-HG (Figure 3(IIIC)). The addition of polyP to the collagen/mucin matrix, followed by incubation with MgCl_2_, leads to an appearance of 50 to 100 nm sized nanoparticles. If those matrices were populated with A549 cells, already a higher density of cells is seen on the polyP-containing hydrogel by eye-inspection (Figure 3(IVA–C)). These qualitative results were substantiated by quantitative MTT analyses (Figure 3V). Again, after 48 h of incubation, an 80% increase in the growth rate is measured on Collagen/mucin-HG in comparison to Collagen-HG. The addition of polyP to the collagen mucin matrix (Collagen/mucin/polyP-HG) even further significantly stimulated growth at 10 µg/mL by 77% and at 100 µg/mL by 45%.

These data indicate that both mucin and polyP, embedded in a collagen hydrogel, stimulate A549 growth significantly.

### 2.6. Effect of PolyP Together with Mucin on Gene Expression of Mucins

**Figure 4:**
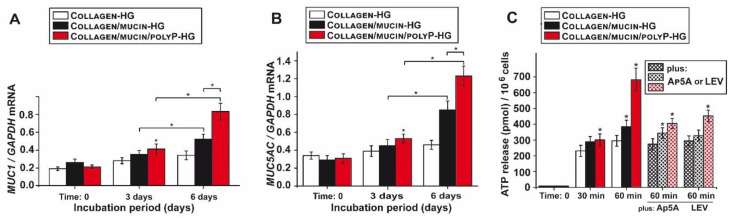
Gene expression level for *MUC1* and *MUC5AC*, and ATP release kinetics in A549 cells in dependence on the three matrices. The expression level (qRT-PCR) for *MUC1* and *MUC5AC* and ATP release kinetics (luciferin–luciferase-based assay) were determined in A549 cells in dependence on the three matrices, Collagen-HG, Collagen/mucin-HG (100 µg/mL mucin), and Collagen/mucin/polyP-HG (100 µg/mL mucin and 10 µg/mL polyP). (**A**) Steady-state expression of *MUC1* in cells cultivated onto the three matrices during the 3 days and 6 days incubation period. (**B**) Expression level of *MUC5AC*. (**C**) Modulation of the ATP release in A549 cells, growing onto the collagen matrix, Collagen-HG, or after addition of mucin (Collagen/mucin-HG) or mucin and polyP (Collagen/mucin/polyP-HG). After an incubation period of 0, 30, and 60 min, aliquots were taken and assayed for the extracellular ATP concentration. For the gene expression studies, RNA was extracted from the cells and then subjected to qRT-PCR. The expression values are correlated to the expression level of the house-keeping gene *GAPDH*. Standard errors of the means (SEM) are indicated (*n* = 5 experiments per time point); * *p* < 0.05. The ATP concentrations were determined in the culture medium (*n* = 10; * *p* < 0.001).

MUC1 is the major mucin type within the periciliary layer forming the respiratory mucus, while MUC5AC is abundantly present in the gel layer overlying the lung epithelium [28]. Therefore, it was consequent to determine the expression level of these two *MUC* species on the gene level in A549 cells. qRT-PCR analyses were performed (Figure 4A,B). During the 6-days incubation period the expression of *MUC1* increases already significantly if the cells are cultivated on Collagen/mucin-HG (100 µg/mL mucin), compared to the expression in cells on Collagen-HG. The addition of polyP to this matrix further increased the steady-state expression level. Interesting is the finding that the gene expression level in cells grown on Collagen/mucin/polyP-HG (100 µg/mL of mucin and 10 µg/mL of polyP) is also significantly higher (by 60%) compared to that cultured on Collagen/mucin-HG (Figure 4A).

A similar expression pattern is also seen in A549 cells with respect to *MUC5AC* (Figure 4B). Additionally, here an initial increase is seen during the first 3 days, but the strong stimulation is measured during the 6-days period. Here again, the expression in cells cultured on Collagen/mucin-HG and also on the Collagen/mucin/polyP-HG is significantly higher compared to Collagen-HG control. Particularly significant is the difference between the expression level in the cells grown for 6 days on Collagen/mucin-HG and the higher level in cells grown on Collagen/mucin/polyP-HG.

### 2.7. Increased Mucin Protein Synthesis After Exposure to PolyP

**Figure 5:**
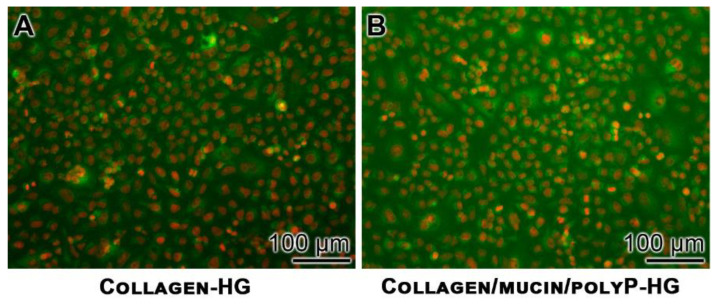
Increased MUC1 synthesis in A549 cells after growing the cells onto the Collagen-HG matrix, in contrast to those growing onto the polyP-containing Collagen/mucin-HG. The immunostaining of the cells grown onto Collagen-HG (**A**) or onto polyP-containing Collagen/mucin-HG (**B)** was performed with anti-MUC1 polyclonal antibodies (green fluorescing), and the nuclei were stained with propidium iodide.

The effect of polyP on the production of MUC1 protein in A549 cells has been determined by immunofluorescence. The human lung cells, A549, were cultured for 72 h and then subjected to an immunostaining procedure with anti-MUC1 polyclonal antibodies. The immunocomplexes were identified with a labelled secondary antibody (staining in green). The nuclei were visualized by counterstaining in red. The images show that the cells that grew on Collagen-HG showed only a small rim or green fluorescence (Figure 5A), while those that had been incubated onto Collagen/mucin/polyP-HG were surrounded by a bulky layer of green fluorescing mucin (Figure 5B).

### 2.8. Increased Release of ATP from A549 Cells in Response to PolyP

ATP is released from alveolar basal epithelial cells, such as A549, after exposure to a series of stimuli in a Ca^2+^-dependent manner [36]. ATP is the most important secretagogue [11] for the surface epithelium of the airway system. Plating A549 cells on Collagen-HG does not affect the ATP export from A549 cells during the 60 min incubation period in vitro (Figure 4C). The addition of mucin to the matrix, Collagen/mucin-HG, caused a slight but significant increase in the ATP release (by 25%) from the cells. However, after the addition of 10 µg/mL of polyP, as in Collagen/mucin/polyP-HG, a sharp induction of ATP release, by 2.3 fold, from the cells is measured. The addition of the ALP inhibitor levamisole (LEV; 1 mM) causes a significant reduction in the ATP release in A549 cells only if these cells are cultured on the polyP-containing matrix. A similar reduction is seen if the cells were preincubated with the ADK inhibitor (P^1^,P^5^-di(adenosine-5′) pentaphosphate (Ap5A; 40 μM). These data strongly suggest that the cell-associated enzymes ADK and ALP, in concert with polyP, are involved in the generation of extracellular ATP.

### 2.9. PolyP PAGE Mobility in the Presence of Mucin

**Figure 6:**
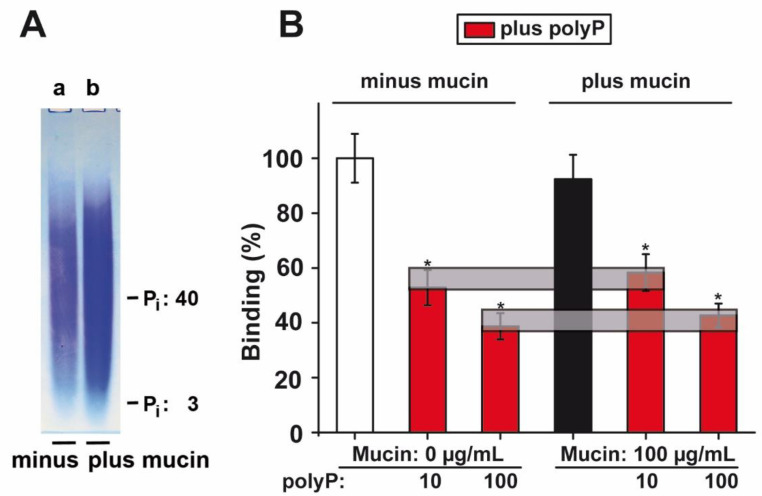
Influence of mucin on the electrophoretic mobility of polyP; effect of polyP on binding of the S-protein receptor-binding domain (RBD) to the ACE2 receptor. (**A**) The PAGE analysis (7 M urea/15% polyacrylamide) of polyP (**a**) without or (**b**) with mucin. (**B**) Influence of polyP in the absence or presence of mucin on the interaction between the viral RBD and the cellular ACE2, as determined by the binding assay. The grey horizontal bars link the corresponding values for the inhibition by polyP in the mucin-free and in the mucin-supplemented assays. Means ± SEM (* *p* < 0.05).

To elucidate, in a first approach, if polyP interacted with mucin, the two components were analyzed by PAGE. In parallel, polyP (100 µg/mL) was size separated by electrophoresis in the absence (Figure 6A-a) or presence of mucin (100 µg/mL) (Figure 6A-b) and then stained with toluidine blue O. Under the conditions used, no effect of mucin on the polyP mobility could be resolved.

### 2.10. Effect of PolyP on Binding of S-Protein to ACE2

PolyP effectively and significantly prevents binding of the RBD of S-protein of SARS-CoV-2 to the ACE2 receptor by 42% at 10 µg/mL (Figure 6B). A further increase in polyP to 100 µg/mL even reduces binding by 57%. This effect has been described already in our first report [49]. For a translational application, it is necessary to clarify if mucin interferes with this effect on RBD:ACE2 binding. It is known that the mucins can associate with other proteins in a hydrophobic manner [60]. The experiments revealed that even in the presence of 100 µg/mL of mucin no effect on the inhibitory activity could be determined (Figure 6B). Therefore, we speculate that the strength of the polyP-caused inhibition might also not be affected under in vivo conditions.

## 3. Discussion

**Figure 7:**
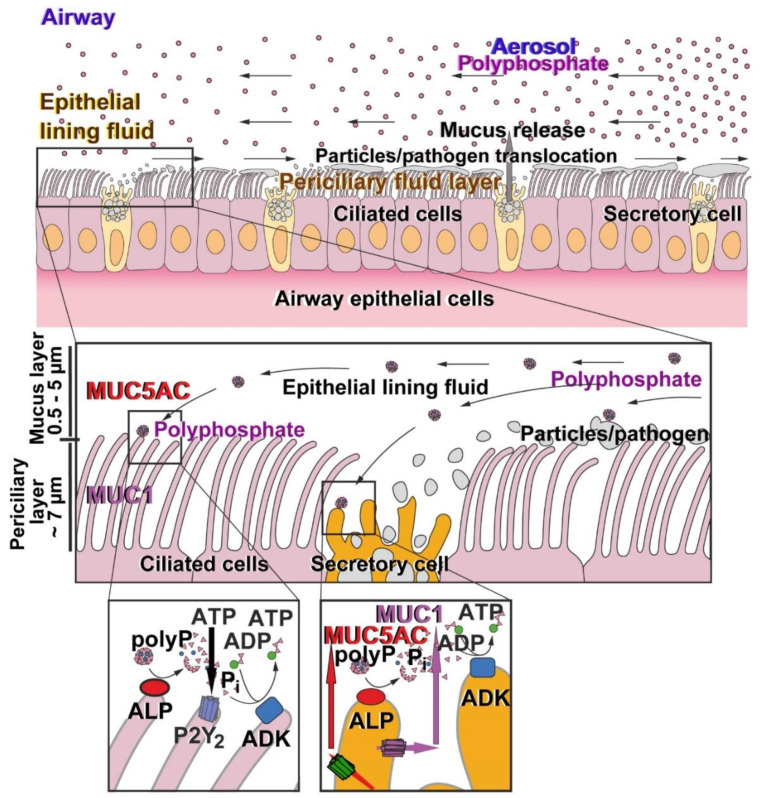
Schematic illustration of the morphogenetic activity of polyP (polyphosphate) on the airway epithelium. The two cells types, the ciliated cells and the secretory cells, compose the airway epithelium. On its surface, both the periciliary fluid layer and the epithelial lining fluid are organized (upper panel). With the directed flow of the mucus the polyP particles are transported both to the secretory and the ciliated cells (middle panel). Both cell types comprise on their outer surfaces the two enzymes, ALP and ADK, which hydrolyze polyP. The liberated free energy is (partially) harvested in the form of ADP (ALP), which subsequently undergoes up-phosphorylation to ATP (ADK). In turn, ATP acts onto the purinergic receptors (P2) eliciting gene induction of *MUC1* and *MUC5AC* followed by a release of these two mucin types.

Surely, airways mucus with its major organic component mucin acts as the first host barrier against inhaled pathogens and, by this, prevents pathogen invasion and the following infection [28]. The adaptation of the mucus to the regionally different mechanical stress is implemented by the array of different mucins and their different mucoadhesive properties, which also control the size-exclusion limits of the mucus against pathogens (<500 nm) [61]; larger particles (>1000 nm) remain trapped on the surface of the mucus. The mucus layer of the airway epithelium is ~100 µm thin and mobile, driven by the ciliated human bronchial cells (Figure 7). The role of the mucins is not restricted to the mechanical barrier function but at least the membrane-bound mucins such as MUC1 also have signaling functions [62]. The intracellular part comprising phosphorylation sites controls the interplay of the different mucins and also forms complexes with E-cadherin and β-catenin, causing a stabilization of cell–cell interactions but also regulated intramembrane proteolysis, followed by a translocation of the cleavage product(s) to the nucleus [63]. The soluble, secreted mucin MUC5AC, which is highly expressed in the lung, plays primarily a barrier function preventing invasion by mucosal pathogens.

Here, we describe that mucin from bovine submaxillary glands causes increased proliferation potential for the alveolar basal epithelial A549 cells. In order to mimic a physiological mucus-like hydrogel environment, the mucin was embedded into collagen, termed Collagen/mucin-HG. After the addition of mucin, the collagen fibrils are glued together, forming a smooth surface. There, cells such as A549 readily attach, followed by a significant induction of the proliferation capacity of the cells.

Two observations prompted us to include polyP as an additional component in the Collagen/mucin-HG. First, polyP is mainly produced in blood platelets, cells that synthesize this polymer in large amounts (reviewed in: [42]) and metabolized via the enzymes ALP and ADK under the formation of ATP in in vitro cultures [33]. This nucleotide is the most important mucus secretagogue for the surface epithelium. Second, the platelets are main producers of the platelet-derived growth factor as well as of the platelet-activating factor that modulate mucin production [64,65]. Until now, polyP has not been determined analytically in physiological airway mucus. The addition of polyP in the presence of MgCl_2_ to the Collagen/mucin-HG matrix, forming Collagen/mucin/polyP-HG, causes the formation of polyP-derived nanoparticles with the same dimensions that have been described previously for the amorphous Mg-polyP nanoparticles [66].

Presented to A549 cells, the polyP component in the Collagen/mucin/polyP-HG strongly stimulates the propensity of the cells not only to attach but also to express the two genes, studied here, of *MUC1* and *MUC5AC*. The increased synthesis of MUC1 was corroborated by immunostaining. Most likely, polyP interacts with the cell membrane inserted receptor for advanced glycation end products (RAGE) as well as the purinergic P2Y_1_ receptor followed by the elicitation of the intracellular Ca^2+^ release [67]. This result in the stimulation of the *MUC* gene expression could have therapeutic impact for corona patients if they treat their dry cough with mucin supplementation, since the MUC1 comprises a major morphogenetically active mucin, is present in airway secretions, and the gel forming MUC5AC controls the characteristic biophysical properties of airway mucus [68], as the lungs’ innate immune defense against inhaled physical, chemical, and pathogenic insults.

Based on existing data [33], it was promising to study if polyP also causes extracellularly an increase in the ATP level, since the airway cells, with A549 studied here, comprise the polyP hydrolyzing enzyme ALP and the metabolic energy generating enzyme ADK as ectoenzymes [47]; Figure 7. It is well established that ATP is released as a co-cargo molecule from mucin-containing granules [69] and stimulates after association with the P2 purinergic receptors the signaling network in epithelial and also in inflammatory cells [70]. The data presented here show that after exposure of polyP to A549 cells the extracellular level of ATP increases strongly. This increase can be abolished by the ADK inhibitor Ap5A and by the ALP inhibitor levamisole [55,56]. Together with earlier results, it is most likely that the extracellular pool of ATP is fueled by polyP [71]. In consequence, polyP and the formed executing nucleotide ATP might act as a powerful physiological metabolite strengthening the innate immunity in general and the anti-viral state in particular, such as in SARS-CoV-2 infection. The interferon activity is reduced in COVID-19 patients [72], a situation that could be normalized and balanced by ATP via the JAK/STAT-1 pathway [73]. ATP reduction as a consequence of SARS-CoV-2, paralleled by pyroptosis, causing the formation of pro-inflammatory cytokines and chemokines [74] might be toned down by ATP supplementation.

The supply of the airway mucus with polyP via the blood platelets is likely in concert with the accumulating inflammatory cells at the airway mucosa [11], but its quantity could be reduced due to the frequently observed thrombocytopenia in COVID-19 patients [48]. The reduced platelet count, which correlates with higher disease severity, is presumably the result of an increased consumption of platelets and/or a decreased production of platelets in the damaged lungs—at least in the Severe Acute Respiratory Syndrome [75].

The function of polyP as a natural and physiological anti-viral polymer acting as a powerful arm in the antiviral defense within the frame of the innate immune system is further supported by the discovery that the polymer acts as a potent inhibitor of the binding of the SARS-CoV-2 S-protein to the cellular ACE2 receptor [49,50]. The binding of polyP to the S-protein has been narrowed down to an interaction of the polymer to the clustered arginine amino acids on the surface of the receptor binding domain of the S-protein. By chemical modification of the arginine units with 1,2-cyclohexanedione a sensitivity of the binding and the resulting inhibition of the S-protein was reached at the physiological polyP concentration of 0.1 µg/mL [50]. In the present study, it could be verified that the inhibitory activity of polyP is not impaired in the presence of mucin. More importantly, through inhibiting the degradation of polyP by the ubiquitous ALP, which is present on the cell surface, including A549, mucin enhances the innate immunity function of the polymer. It can be assumed that mucin also potentiates the antiviral and morphogenetic effects of polyP against other virus infections. An antiviral effect of polyP has also been observed against the human immunodeficiency virus 1 (HIV-1 [76]). In addition, an inhibitory effect of mucins has been proposed for this virus [77]. It is likely that the dual activities of polyP against viral infections on the level of virus host cell interaction and innate immunity is not limited to SARS-CoV-2 but also plays a general role in the natural antiviral defense of cells/tissues.

## 4. Materials and Methods

### 4.1. Materials

Na-polyphosphate (Na-polyP) with an average chain length of 40 P_i_ units (polyP_40_) was from Chemische Fabrik Budenheim (Budenheim; Germany). In addition, the following materials were purchased: levamisole hydrochloride (LEV; #L9756 Sigma, Taufkirchen; Germany), Ap5A (P^1^,P^5^-di(adenosine-5′) pentaphosphate pentasodium salt; #D4022 Sigma, Taufkirchen; Germany), alkaline phosphatase (calf intestine, ≥1500 units/mg; #524572 Sigma, Taufkirchen; Germany), mucin from bovine submaxillary glands (type I-S prepared according to [78]) (#M3895 Sigma, Taufkirchen; Germany), and HEPES (*N*-(2-hydroxyethyl)piperazine-*N*′-(2-ethanesulfonic acid); #H3375 Sigma, Taufkirchen; Germany). Collagen isolated from bovine tendon collagen type I and dissolved in 0.1 M acetic acid (at pH 3.6) was a gift of Lando Biomaterials (Shenzhen; China).

### 4.2. Alkaline Phosphatase Assay and PolyP PAGE

The alkaline phosphatase (calf intestine, ≥1500 units/mg; #524572 Sigma, Taufkirchen; Germany) was dissolved in a 10 mM Tris-HCl buffer (pH 8.0; 1 mM MgCl_2_, 0.1 mM ZnCl_2_, 50 mM KCl). In a total volume of 500 µL, the enzyme was added at a concentration of 5 µg/mL to the incubation assay together with 100 µg/mL of Na-polyP and incubated at 37 °C for 60 min. Then, aliquots of 20 µL were taken and analyzed by gel electrophoresis. PolyP was incubated either in the absence or presence of mucin (100 µg/mL).

After incubation, polyP aliquots were resolved by polyacrylamide gel electrophoresis (PAGE) using 20% polyacrylamide gels (acrylamide 29:1 bisacrylamide; 60 mm height) containing 7 M urea in TBE buffer (Tris-borate/EDTA (100 mM-100 mM/2 mM)) pH 8.3, as described [79,80]. Then, the gels were stained with toluidine blue O (#198161 Sigma, Taufkirchen, Germany), where indicated mucin (100 µg/mL) was added to the polyP (100 µg/mL) prior to the loading onto the gel.

### 4.3. Cells

A549 cells (#86012804 Sigma, Taufkirchen, Germany), a human lung (carcinoma) line, were grown in Ham’s F-12K (Kaighn’s) medium (#21127022; Gibco/Thermo Fisher Scientific, Dreieich, Germany), supplemented with 10% fetal bovine serum (FBS), 1% penicillin-streptomycin, and 4 mM glutamine. The cells were incubated in 96-well plates/24-well plates in a humidified atmosphere of 5% CO_2_ in air (37 °C).

### 4.4. Incubation of A549 Cells on Collagen–Collagen/Mucin–Collagen/Mucin/PolyP Hydrogel

The A549 cells were seeded onto either collagen hydrogel, collagen/mucin hydrogel, or collagen/mucin/polyP hydrogel.

Collagen hydrogel (Collagen-HG): Bovine collagen was dissolved in 0.1 M acetic acid (pH 3.6) at a concentration of 10 mg/mL. After adjusting the pH to 7.4 (with NaOH), the solution was brought to 5 mg/mL and supplemented with 10 mM MgCl_2_ (#M1028 Sigma); Collagen-HG. Then, 50 µL or 300 µL of the solution were added per well (96-well/24-well plates). After standing in the incubation chamber for 6 h, the plates were washed 3 times with phosphate buffered saline (PBS), followed by a second washing cycle conducted 3 times with medium. Then, the plates were overlayed with 100 µL (96-well plates) or 500 µL (24-well plates) of cell suspension (20 × 10^3^ cells/mL). In the third PBS washing solution, the Mg^2+^ concentration was determined by complexometric titration [81]. Less than 1 mM Mg^2+^ was detected.

Collagen/mucin hydrogel (Collagen/mucin-HG): An aliquot of 1 mL of collagen solution (pH 3.6) was supplemented with 1 mL of mucin (concentration between 0 and 1 mg/mL (final); average concentration in the human saliva ~200 µg/mL [82]) and the mixture was brought to 10 mM MgCl_2_; Collagen/mucin-HG. The pH was adjusted to 7.4 (with NaOH), and the bubbles were removed by centrifugation. Then, the mixture was layered into the well plates and overlayed with the cells.

Collagen/mucin/polyP hydrogel (Collagen/mucin/polyP-HG): In the last series the collagen–mucin (200 µg/mL) mixture was supplemented with Na-polyP (concentration range of 0 to 100 µg/mL (final)). MgCl_2_ was stoichiometrically added (molar ratio of 2 (with respect to phosphate monomer) to 1 [Mg^2+^]) to compensate for any effect caused by the chelating activity of polyP [83]; Collagen/mucin/polyP-HG. The content of polyP was quantitated by using an Ultra Micro Balance (Mettler-Toledo, Gießen, Germany). Finally, the cells were added.

The cells were stained by calcein pretreatment (#17783 Sigma) [84].

### 4.5. Fourier-Transform Infrared Spectroscopy from the Hydrogel *Samples*

Samples of Collagen/mucin-HG, Collagen/mucin/polyP-HG, and as reference also from Mg-polyP nanoparticles (Mg-polyP-NP) were analyzed by Fourier-transform infrared spectroscopy (FT-IR) using an attenuated total reflectance-FTIR spectroscope/Varian IR spectrometer (Agilent, Santa Clara, CA, USA). The Mg-polyP-NP was prepared from Na-polyP and MgCl_2_ [66].

### 4.6. Cell Viability Studies

Cell proliferation was evaluated with the MTT (3-(4,5-methylthiazol-2-yl)-2,5-diphenyl-tetrazolium bromide; # M6494, Thermo Fisher Scientific) assay. In short, 200 µL of 1 × 10^4^ A549 cells were seeded into 96-well plates, coated with the respective hydrogel, and kept there overnight. Then, the medium was replaced with fresh medium, and the cells were grown for an additional 48 h on the hydrogel. Finally, 50 μL of MTT (10 mg/mL) were added into each well and 2 h later the solubilization buffer (10% SDS with 0.01 N HCl) was added. After incubation overnight, the intensity of the developed color was determined at 550 nm in a microplate reader (Bio-Rad Laboratories, Feldkirchen, Germany).

### 4.7. Immunofluorescence Studies

A549 cells were seeded either onto the plain collagen hydrogel, or onto the collagen/mucin/polyP hydrogel in 24-well plates (addition of 500 µL of a 20 × 10^3^ cells/mL suspension). After an incubation period of 72 h, the cells were fixed (3.7% formaldehyde; 20 min) and permeabilized (0.1% Triton X-100 for 80 min). After blocking with 10% normal goat serum, the cells were interacted with anti-MUC1 (#PA5-81524; Thermo Fisher Scientific; dilution 1:1000) at 4° C overnight. Then, the cells were incubated with Alexa Fluor 488-conjugated goat anti-rabbit IgG secondary antibody (green). The cells were counterstained with propidium iodide (#P4864; Sigma-Aldrich) allowing the nuclei to light up in red. Images were taken with a fluorescence microscope (Olympus, Hamburg, Germany).

### 4.8. Determination of Extracellular ATP Concentration

The release of ATP was quantified by applying the luciferin–luciferase-based Enlighten assay (Promega, Madison, WI, USA) according to the manufacturer’s instructions and as described [44]. Briefly, the A549 cells were grown in 24-well plates to 90–100% confluency. Then, the cells were transferred into Ham’s medium without serum, and incubation was continued for additional 30 or 60 min at the cell density of 10^6^ cells/mL. The following matrices were used for cell cultivation; Collagen-HG, Collagen/mucin-HG (100 µg/mL of mucin), or Collagen/mucin/polyP-HG (100 µg/mL of mucin and 10 µg/mL of polyP). Subsequently, a sample of 0.5 mL was collected and transferred into chilled polypropylene tubes (#Z334006 Sigma) and centrifuged (12,000× *g*; 5 min; EBA 200; Hettich GmbH, Tuttlingen, Germany). Aliquots (100 μL) were taken from the supernatant and measured in the luciferin–luciferase assay. From the standard curve, the ATP level was read. The ATP concentrations are given as pmol/10^6^ cells. Where mentioned, the cells were pre-incubated with 40 μM Ap5A (inhibitor of ADK) or 1 mM of LEV (inhibitor of the ALP) for 10 min prior to cell seeding.

### 4.9. Quantitative Real-Time Polymerase Chain Reaction

The qRT-PCR was performed as described [85]. In brief, A549 cells were incubated either onto Collagen hydrogel, Collagen/mucin hydrogel, or onto Collagen/mucin/polyP hydrogel for 3 or 6 days. After termination, RNA was extracted and subjected to qRT-PCR analysis using the following primer pairs [31]: For human *MUC1*, expressed in the periciliary layer [28], (Accession number; P15941) Fwd: 5′-AATTGACTCTGGCCTTCCGA-3′ and Rev: 5′-TGCCACCATTACCTGCAGAA-3′, and for human *MUC5AC*, strongly expressed in the airways [11], (U06711) Fwd: 5′-TCCGGCCTCATCTTCTCC-3′ and Rev: 5′-ACTTGGGCACTGGTGCTG-3′. The expression levels determined were correlated with the one of the reference housekeeping gene *GAPDH* (glyceraldehyde 3-phosphate dehydrogenase; NM_002046.3) with the primer pair Fwd: 5′-ACTTTGTGAAGCTCATTTCC-3′ and Rev: 5′-TTGCTGGGGCTGGTGGTCCA-3′. The analysis (in triplicate) was performed in an iCycler (Bio-Rad, Hercules, CA; USA). The mean C_t_ values and efficiencies were calculated by the iCycler software (Bio-Rad, Hercules, CA; USA) [86]; the estimated PCR efficiencies were 95–103%.

### 4.10. Inhibition of Binding of SARS-CoV-2 S-Protein to ACE2

The Screening Assay Kit (BPS Bioscience/Tebu-bio, Offenbach, Germany) for the determination of the binding of the RBD of the viral S-protein to the ACE2 cell surface receptor was used. With this assay the effect of polyP on the strength of interaction between the RBD and the ACE2 was quantitated. The ACE2 (50 ng/well) was bound to the bottom of the 96 well plate and then allowed to interact with the RBD/S1-protein (100 ng/well), labeled with biotin. The complex S-protein with ACE2 was detected with streptavidin–horseradish peroxidase (HRP) using the HRP substrate. The inhibitor, polyP, was added to the RBD. After a preincubation period of 60 min (23 °C) in 10 mM HEPES buffer, pH 7.0, the RBD was exposed to the ACE2. In the experiments with mucin, the protein was added for 60 min at a concentration of 100 µg/mL (dissolved in 10 mM HEPES) to the ACE2, followed by three washing cycles with 10 mM HEPES buffer (pH 7). The assay was, if not mentioned otherwise, performed at 23 °C. After blocking/washing, the samples were measured. The chemiluminescence was quantitated with a Perkin Elmer-Wallac victor 3V multi-label microplate reader (Perkin-Elmer, Waltham, MA, USA). The values for the blank (immuno buffers, loosely bound components in the system and buffer used for dissolving the polyP) were subtracted from the readings. The values obtained for the samples without inhibitor served as reference and were set to 100%.

### 4.11. Microscopic Analyses

For the scanning electron microscopic (SEM) inspections, the HITACHI SU8000 microscope (Hitachi, Krefeld, Germany) was used. The light microscopical images were taken with a VHX-600 Digital Microscope (Keyence, Neu-Isenburg, Germany) or with the Olympus fluorescence microscope (Olympus, Hamburg, Germany) using the wavelengths 496 nm (excitation) and 520 nm (emission) for calcein stained samples.

### 4.12. Statistical Analysis

The values represent the respective average ± standard deviations (σ). Student *t* test was applied to perform comparisons between two groups. Usually, the average values and σ originated from at least three to six independent experiments. Values of *p* < 0.05 were considered statistically significant (*). The calculations were performed with the GraphPad Prism 7.0 software (GraphPad Software, La Jolla, CA, USA).

## 5. Conclusions

At least for the transition period until a vaccine against SARS-CoV-2 has been developed, a control of the COVID-19 disease is restricted to powerful antiviral compounds and to strengthening the innate immune system. The innate immunity arsenal comprises two effective arms; first, protective, physical—physiological—biochemical barriers against infection and second, cell-driven defense systems, which rely on engulfment, phagocytosis, and intracellular killing. The first defense system that limits entry and control invasion of foreign pathogens is the skin with the mucous membranes and fluids. The surface mucus provides a size-dependent protective shield of mucins with different compositions, adapting to a given situation with a changing profile of mucins and directed to pathogenic bacteria and viruses and also with a suitable viscosity trapping them. The adaptive composition of the mucus is adjusted by secreting cells via an elaborated ligand receptor system, mainly dependent on the interaction of ATP with the airway epithelial purinergic receptors.

It is hoped that parallel with the stimulated/increased level of the mucins in response to polyP exposure, an induced secretion of lactoferrin and lysozyme by the airways epithelial cells will also be observed [87]. Such an accumulation of the two most abundant antimicrobial proteins in the airway mucus might even function as a breaking open agent against hard/resistant viral shells [24]. The polyP-based scaffold can be readily linked via ionic bonds in the presence or absence of Ca^2+^ with mucins to utilize the manifold antimicrobial properties of the mucins [41].

While ATP has previously been considered to originate exclusively from intracellular sources such as the mitochondrial respiratory chain, the present study provides evidence that the extracellular generation of ATP by enzymatic hydrolysis of polyP via ALP and the connected up-phosphorylation of ADP to ATP with the enzyme ADK also contributes to the extracellular ATP (Figure 7). Most likely, polyP interacts with the cell membrane inserted receptor for advanced glycation end products (RAGE) as well as the purinergic P2Y_1_ receptor followed by the elicitation of the intracellular Ca^2+^ release [67]. This result on the stimulation of the *MUC* gene expression could have a therapeutic impact for corona patients if they treat their dry cough with mucin supplementation, e.g., MUC1. In addition, polyP in the form of Ca-polyP-NP is taken up by the cells and undergoes a dissolution and hydrolysis of polyP by the ALP, followed by a conversion of the released energy in metabolically useful energy/acid anhydride linkage in ATP [44].

It remains to be studied if the efficiency of this ATP generation is impaired during SARS-CoV-2 infection. If so, therapeutic substitution with polyP might be indicated because this polymer can be synthesized straightforwardly and is in limits not toxic and not immunogenic (see: [88]). This effect of polyP can be considered to be effective against a range of respiratory viruses that interact with airway epithelial cells. A second more specific interaction of polyP has been disclosed. The latter effect is based on a specific inhibitory interaction of polyP with the S-protein of SARS-CoV-2. Since the polyP metabolic systems are already present before the manifestation of the infection, this defensive molecule is also a part of the innate immunity against the virus.

## Abbreviations

2019-nCoV	Novel coronavirus
ACE2	Angiotensin-converting enzyme 2
ADK	Adenylate kinase
ADP	Adenosine diphosphate
ALP	Alkaline phosphatase
Ap5A	P^1^,P^5^-di(adenosine-5′ pentaphosphate pentasodium salt
ATP	Adenosine triphosphate
Collagen/mucin/polyP-HG	Collagen/mucin/polyP-hydrogel
Collagen/mucin-HG	Collagen/mucin hydrogel
Collagen-HG	Collagen hydrogel
FBS	Fetal bovine serum
FT-IR	Fourier-transform infrared spectroscopy
HEPES	*N*-(2-hydroxyethyl)piperazine-*N*′-(2-ethanesulfonic acid)
LEV	Levamisole hydrochloride
Mg-polyP-NP	Mg-polyP nanoparticles
MTT	3-[4,5-methylthiazol-2-yl]-2,5-diphenyl-tetrazolium bromide
Na-polyP	Na-polyphosphate
PAGE	Polyacrylamide gel electrophoresis
polyP	Polyphosphate
polyP_40_	Polyphosphate with an average chain length of 40 P_i_ units
qRT-PCR	Quantitative real-time polymerase chain reaction
RBD	Receptor-binding domain
SARS-CoV-2	Severe acute respiratory syndrome coronavirus 2
SEM	Scanning electron microscopy
TBE buffer	Tris-borate/EDTA buffer
